# Self-Disinfecting Urethral Catheter to Overcome Urinary Infections: From Antimicrobial Photodynamic Action to Antibacterial Biochemical Entities

**DOI:** 10.3390/microorganisms10122484

**Published:** 2022-12-15

**Authors:** Lucas D. Dias, Luana S. Duarte, Plínio L. F. Naves, Hamilton B. Napolitano, Vanderlei S. Bagnato

**Affiliations:** 1 Grupo de Química Teórica e Estrutural de Anápolis, Universidade Estadual de Goiás, Anápolis 75132-903, GO, Brazil; 2 Laboratório de Novos Materiais, Universidade Evangélica de Goiás, Anápolis 75083-515, GO, Brazil; 3 São Carlos Institute of Physics, University of São Paulo, São Carlos 13560-970, SP, Brazil; 4 Department of Chemical Engineering, Federal University of São Carlos, São Carlos 13565-905, SP, Brazil; 5 Laboratório de Bioensaios, Universidade Estadual de Goiás, Anápolis 75132-903, GO, Brazil; 6 Department of Biomedical Engineering, Texas A&M University, College Station, TX 77843, USA

**Keywords:** infections, medical device, urinary catheter, antimicrobial coating, photodynamic therapy, antimicrobial drugs

## Abstract

Medical-device-related infections are considered a worldwide public health problem. In particular, urinary catheters are responsible for 75% of cases of hospital urinary infections (a mortality rate of 2.3%) and present a high cost for public and private health systems. Some actions have been performed and described aiming to avoid it, including clinical guidelines for catheterization procedure, antibiotic prophylaxis, and use of antimicrobial coated-urinary catheters. In this review paper, we present and discuss the functionalization of urinary catheters surfaces with antimicrobial entities (e.g., photosensitizers, antibiotics, polymers, silver salts, oxides, bacteriophage, and enzymes) highlighting the immobilization of photosensitizing molecules for antimicrobial photodynamic applications. Moreover, the characterization techniques and (photo)antimicrobial effects of the coated-urinary catheters are described and discussed. We highlight the most significant examples in the last decade (2011–2021) concerning the antimicrobial coated-urinary catheter and their potential use, limitations, and future perspectives.

## 1. Introduction

Health-care associated infections (HAIs) lead to an increase in morbidity and financial burden, and in some cases, resulting in death [1,2]. Among them, infections associated with medical devices (e.g., intravenous lines, endotracheal tubes, central venous catheters, urinary catheter and others) are responsible for approximately 70% of cases of nosocomial infections resulting in complications that patients need even more medical protocols including the use of antibiotic therapy (contributing to microbial resistance to clinical antibiotics) [3,4,5].

The medical devices are widely used in routine medical procedures, increasing life expectancy, providing a better stay for patients in hospitals and facilitating their recovery [4,6]. Herein, we highlight the urinary catheters (temporary or long-term use) which are extremely applied (20% and 61% in non-intensive and intensive care units, respectively) [1] for the treatment and relief of hospitalized patients or those with diseases that need constant use [7,8]. The urinary catheter are made by different polymers (such as silicone, polyvinyl chloride (PVC), polyurethane (PU), latex rubber) and present the function of draining urine from the bladder [9], being used in cases of debilitated, paralyzed or comatose patients, presenting incontinence and urinary retention [2] or in anesthetized or sedated patients in surgical procedures [1].

**Figure 1:**
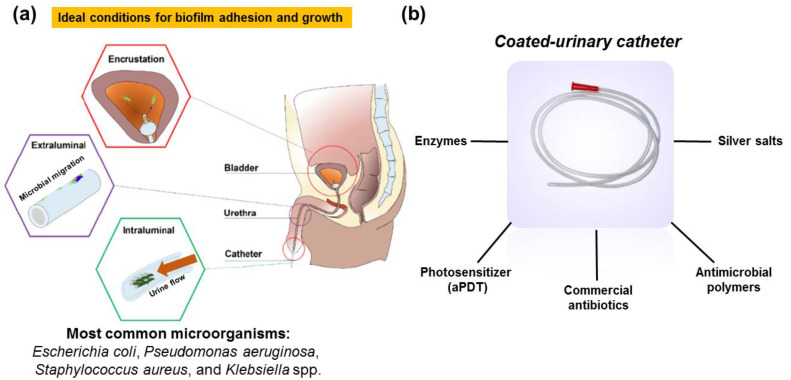
(**a**) Pathogenesis of CAUTIs; (**b**) examples of antimicrobial coated-urinary catheter reported so far. Adapted from [23] (open access). Copyright 2021 MDPI.

Besides the urinary catheters benefits, its use is precursor to more than 75% of cases of hospital urinary infections [10,11] and responsible for up to 40% of nosocomial infections showing a mortality rate of 2.3% [12]. Catheter-associated urinary tract infection (CAUTIs) occur during insertion of the probe into the urinary canal, external bacteria can pass through the lumen of the catheter or through the outside of the catheter from the urethra to the bladder [13]. Moreover, on the outside surface of the catheter, the biofilm formation begins via adhesion of pathogenic bacteria (i.e., *Escherichia coli*, *Staphylococcus aureu*, *Pseudomonas aeruginosa*, *Klebsiella* spp., and others) and/or fungi (e.g., *Candida* spp.) to catheter surface but it dependent on the hydrophobicity of the bacteria and the catheter [4]. It is important to mention that biofilm is a complex polymeric matrix formed by exopolysaccharides, proteins, and microbes that attach themselves to a surface, making it more difficult to eradicate [14,15] (Figure 1a).

Nowadays, in order to avoid a greater number of CAUTIs, there are clinical guidelines for the cases in which the probe should be used in hospital routine. It is recommended to apply only for specific cases, using for the time strictly necessary and also following aseptic catheter insertion technique (e.g., training, hand hygiene, adequate lubricant, and with smallest caliber catheter) [1,16,17]. Additionally, antibiotic prophylaxis has been largely performed, however, these strategies have not demonstrated sufficient efficiency due to the presence of multiresistant bacteria.

In this regard, several efforts have been made to propose a viable and efficient alternative to fight this nosocomial infection namely functionalization of urinary catheter surface with antimicrobial entities e.g., photosensitizers (for antimicrobial photodynamic therapy-aPDT) [18], antibiotics [19,20], polymers [21], silver salts [22], and others have been reported (Figure 1b). These antimicrobial catheters aim to avoid biofilm formation (including adhesion) and also biofilm eradication during the medical procedures.

Beside the complex composition of biofilm (proteins, extracellular DNA, enzymes, lipid, epithelial cells, and others) [17], the attachment of bacteria to a surface rapidly alters the expression of several genes responsible for the fabrication and maturation of extracellular exopolysaccharides, which results in a protective barrier against external chemical agents or the host’s defense system. In addition, the biofilm protects adherent bacteria from mechanical/physical stress, antimicrobial action, and the host’s immune defenses [24]. Herein, we analyze the past decade (2011–2021) concerning the functionalization and characterization of urinary catheter surfaces with antimicrobial entities namely photosensitizers (for aPDT), antibiotics, polymers, silver salts, oxides, bacteriophage, and enzymes. We present the most significant and illustrative examples for this period. For each example, we report in detail the functionalization processes, antimicrobial entities, characterization techniques, microorganisms used as well as their antimicrobial efficiency.

## 2. Functionalization of Urinary Catheter Surfaces with Antimicrobial Entities

### 2.1. Photosensitizing Molecules

Antimicrobial photodynamic therapy is an alternative and efficient tool able to photoinactivate pathogenic microorganisms such as bacteria, protozoa, fungi, and virus as demonstrated in several in vitro [25], in vivo [25] and clinical trials studies [26]. Due to its multi-target mode of action (unlike antimicrobial drugs), aPDT is described as a powerful tool showing a low probability to develop photoresistent strains and also able to kill antibiotic resistant bacteria [27].

**Figure 2:**
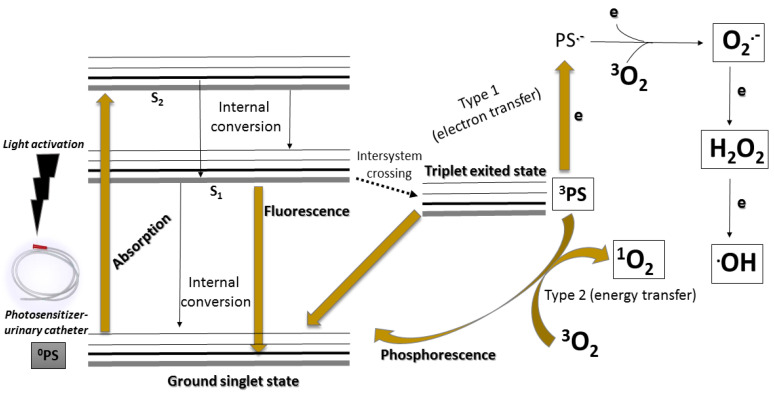
Simplified Jablunski diagram that present the general mechanism of action of the aPDT.

Concerning its mechanism, aPDT acts based on a combination of a photosensitizing molecule (PS), molecular oxygen (O_2_), and a light source with appropriate wavelength with the purpose of produce reactive oxygen species (ROS) followed by oxidation of biomolecules present on pathogenic microorganisms. This mechanism of action can undergo two pathways and generate two groups of ROS: (i) H_2_O_2_ (hydrogen peroxide); O_2_^−^ (superperoxide anion); OH (hydroxyl radical) (Type I mechanism); (ii) ^1^O_2_ (singlet oxygen) (type II mechanism), as described in Figure 2 [28].

**Figure 3:**
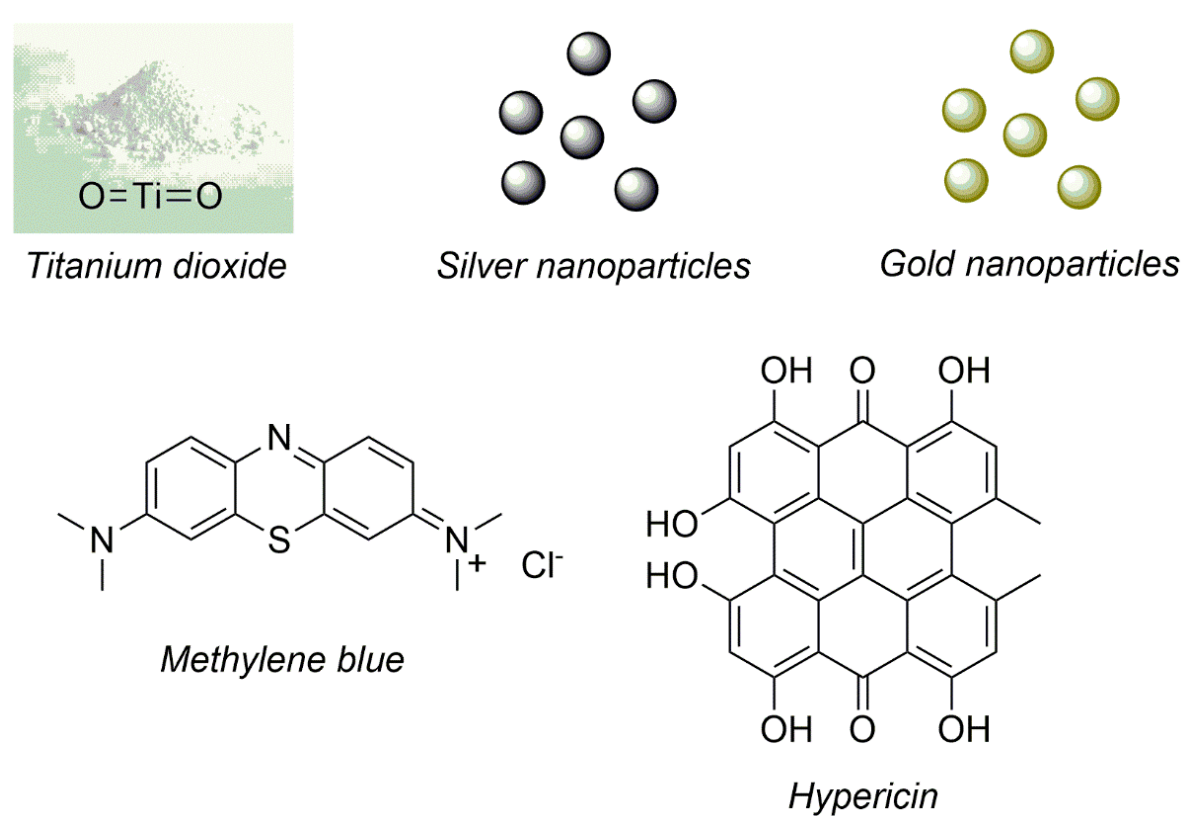
Photosensitizers immobilized onto urinary catheters and described in this work.

In this regard, as a result of the high efficiency and low toxicity of aPDT, there are some studies which reported the functionalization of the urinary catheter surface with photosensitizers (organic and inorganics) [29]. This functionalization aims to produce an antimicrobial photodynamic action on functionalized-urinary catheter surface when illuminated avoiding the presence of pathogenic microorganisms and growth of biofilm [30]. Moreover, beyond the antimicrobial photodynamic action, the urinary catheter should maintain its ideal physicochemical (wettability and roughness) and mechanical (stiffness and flexibility) properties after the functionalization process in order to produce the exact clinical function. Figure 3 shows the 2D molecular structures of photosensitizers immobilized onto urinary catheters that are described in this work.

In 2015, Bovis and collaborators described the incorporation of methylene blue and gold nanoparticles (AuNPs) on silicone urinary catheter surface by a simple methodology (swell–encapsulation–shrink’ technique) [31]. For that, the authors added AuNP and methylene blue (at 700 mg L^−1^) in acetone during 24 h. This functionalized urinary catheter was characterized by transmission electron microscopy (TEM), fluorescence microscopy, fiber optic confocal laser endomicroscopy, time-resolved EPR spectroscopy, continuous-wave electron paramagnetic resonance (EPR) spectroscopy, time-resolved ^1^O_2_ phosphorescence detection, ^1^O_2_ measurements with sensor green probe, and O_2_ consumption during laser irradiation. Furthermore, the functionalized silicone urinary catheter (segments) presented 3 log_10_ of photoreduction using *Staphylococcus epidermidis* bacteria as a bacteria model under illumination (at 660 nm, 45 J/cm^2^). The authors also reported that the prior sterilization of functionalized urinary catheter with ethylene oxide did not modify the photoantibacterial effect. 

In 2019, Amaro and co-authors presented at the 17th International Photodynamic Association World Congress (MA, United States) a covalent functionalization process of silicone urinary catheter with a derivative metalloporphyrin (the exact molecular structure of the derivative metalloporphyrin was not described by the authors) [18]. Initially, the authors functionalized the urinary catheter with amine groups using 3-aminopropyltriethoxysilane (APTES) or 3-aminopropyltrimethoxysilane (APTMS) followed by reaction with the derivative metalloporphyrin. The metalloporphyrin-functionalized urinary catheter showed a considerable photoantibacterial effect against *P. aeruginosa* and *S. epidermidis* (inactivation up to 99.9%) under illumination (at 532 nm, 100 mW, during 30 and 60 min).

In 2019, Vögeling and co-authors evaluated the photoantibacterial action of a modified-polyurethane catheter [32]. The catheter surface was functionalized by inclusion of a complex (hypericin and 2-hydroxypropyl)-β-cyclodextrin) or a liposomal particle containing hypericin (a natural anthraquinone) as photosensitizer using layer by layer approach. This photoactivated-catheter was characterized by scanning electron microscope (SEM), atomic force microscopy (AFM) and the concentration of hypericin on its surface was also determined. Its photoantibacterial effect was evaluated against *Staphylococcus saprophyticus* subsp. *bovis* (DSM 18669) biofilm presenting a reduction of 4.3 log_10_ and improved by addition of ultrasound (bacterial reduction of 6.8 log_10_). In this regard, ultrasound has been described as an antimicrobial tool through activation of a sonosensitizer. Its mechanism is based on inertial cavitation produced by collapsing microbubbles and formation of free radicals [33].

The functionalization of photosensitizers onto urinary catheters shows as a promising tool to photoinactivate pathogenic microorganisms onto catheter surfaces. At the last years, few photosensitizers (such as methylene blue, gold nanoparticles, and silver nanoparticles) were evaluated without any systematic evaluation. Other classes of photosensitizers (e.g., porphyrin, chlorin, bacteriochlorin, and phthalocyanines) should be tested as well as their in vitro/in vivo safety studies. Moreover, the light source device should be designed aiming at the extracorporeal illumination of the urinary tract.

### 2.2. Antibiotics and Antimicrobial Agent

**Figure 4:**
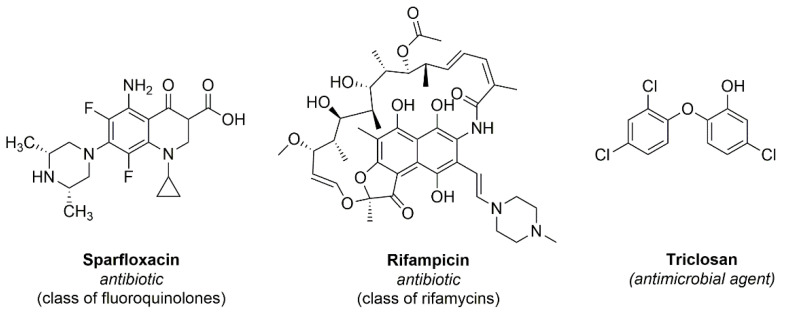
Antibiotics and an antimicrobial agent immobilized onto urinary catheters and described in this work.

Antibiotics are natural, semi-synthetic or synthetic compounds that interfere with the development of bacteria, inhibiting their growth or even causing their death aiming to fight bacterial infections [34]. These are divided into classes, such as phosphomycin, nitrofurantoins, sulfonamides and beta-lactams, examples of antibiotics most used in cases of urinary infections [35]. In order to develop antibacterial urinary catheters, some authors described the immobilization of commercial antibiotics onto urinary catheters. This functionalization shows the same goal of the aPDT, in other words, to promote the inhibition of biofilm and pathogenic bacteria on the catheter surface. Figure 4 describes the 2D molecular structures of antibiotics (sparfloxacin and rifampicin) and an antimicrobial agent (triclosan) immobilized onto urinary catheters described and discussed in this work.

Table 1 shows a description of the functionalized urinary catheters with commercial antibiotics (2011–2021) and their evaluation parameters used for characterization, bacteria applied, and their efficiency.

**Figure 5:**
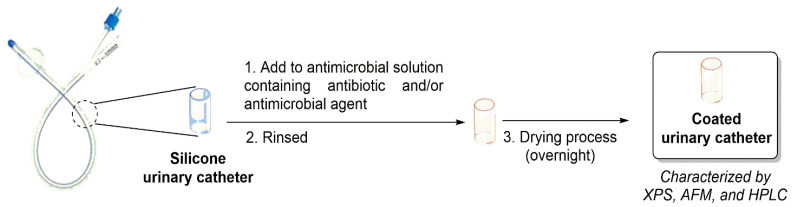
General impregnation process of urinary catheter with antibiotics and antimicrobial agent.

Fischer and co-authors (in 2015) (Table 1, entry 1) [36] reported the impregnation of of antibiotics (e.g., rifampicin and sparfloxacin) and an antimicrobial agent (triclosan) on silicone-based urinary catheters and their evaluation against *E. coli*, *P. mirabilis*, *K. pneumoniae*, *E. faecalis*, and *S. saprophyticus* from clinical isolates from patients with CAUTIs (Figure 5). To analyze the functionalization processes applied, these antibiotic impregnated-urinary catheters were analyzed by XPS, AFM, and HPLC. Additionally, the release of the antibiotics from the functionalized urinary catheters were analyzed by HPLC and the following results were obtained: rifampicin was not detected until 28 days; 5.9–33.4% for triclosan and 11.1–44.3% for sparfloxacin. Furthermore, the authors evaluated the antimicrobial activity of these commercial antibiotics (e.g., rifampicin, triclosan, and sparfloxacin) on the urinary catheter surfaces and observed that no viable *S. aureus*, *S. saprophyticus* or *K. pneumoniae* were present after 24 h. Moreover, *E. coli* was completely killed after 48 h and *E. faecalis* in 72 h. *P. mirabilis* suffered a reduction of 99.9% after 72 h. 

In 2019, Belfield and co-authors [19] (Table 1, entry 2) evaluated an antimicrobial urinary catheter impregnated with a group of antibiotics (rifampicin (0.080% w/w), triclosan (1.084% w/w), and sparfloxacin (0.704% w/w)) against beta-lactamase producing *E. coli*, and carbapenemase-producing *E. coli*, *P. mirabilis*, *S. saprophyticus*, and *E. coli* collected from ureteral stents and indwelling urinary catheters. The functionalized urinary catheters were characterized by AFM, XPS, TEM, static model of encrustation, in vitro flow model of encrustation, SEM, and in vitro flow challenge model. The results demonstrated that the roughness of the catheter surface after the functionalization methodology was not altered. The authors observed that the antimicrobial urinary catheter inhibited for 12 weeks the establishment by methicillin-resistant *S. epidermidis*, MRSA, carbapenemase-producing *E. coli*, and extended-spectrum beta-lactamase producing *E. coli*.

In 2020, Burroughs and co-authors [20] (Table 1, entry 3) reported two different strategies for preventing bacterial biofilm attachment and formation onto urinary catheter surfaces. The authors performed the coating of silicone catheter with polyacrylate (polymerization reaction) impregnated with rifampicin, sparfloxacin and triclosan antibiotics using solutions with concentrations of 0.2%, 1.0%, and 1.0%, respectively. Besides, these coated-catheters were evaluated against *E. coli* and *S. aureus* bacteria (obtained from patients with CAUTI) showing a bacteria reduction and biofilm formation prevention. In order to characterize the functionalized urinary catheters, ToF-SIMS and SEM tests were carried out. The authors observed that the polyacrylate coatings did not kill planktonic bacteria, which is consistent with the mechanism of action of the polyacrylate acting specifically on biofilm formation prevention by an electrostatic interaction between the polymer and the biofilm matrix.

Overall, the use of antibiotics onto catheters is useful antimicrobial strategy, however the development of bacteria resistance should be considered and analyzed before their use on a large scale. Moreover, the leaching of the antibiotics and antimicrobial agents from the catheters should be analyzed and described.

### 2.3. Antimicrobial Polymers

Antimicrobial polymers (natural, semi-synthetic or synthetic) are agents whose surface hinders bacterial adhesion and, consequently, the biofilm formation [37]. These polymers also act by damaging the cell membrane of bacteria and also can release (delivery systems) antibiotics, nanoparticles, and other chemical or biological entities. Table 2 shows a description of antimicrobial coatings for urinary catheters (2011–2021) presenting their parameters applied for characterization, microorganisms, and efficiency.

In 2019, Matej and co-authors [38] (Table 2, Entry 1) immobilized colloidal polysaccharides (chitosan derivatives and hyaluronic acid combined with a lysine-based surfactant) onto silicone to create an antimicrobial coating. These polymers were tested against *E. coli*, *Methicillin resistant S. aureus* (MRSA), *S. aureus*, F+ conjugated *E. coli*, and *Candida albicans*. The materials surface were characterized by FTIR, XPS, CLSM and SEM. The most effective process to build the coating of polydimethylsiloxane with colloidal polysaccharide complex was the discontinuous 3-step dip-coating process. The developed coating demonstrated an inhibition of bacterial growth up to 86% and the hyaluronic acid surfactant-based coating also displayed a significant biofilm prevention.

In 2020, Alves and co-authors [39] (Table 2, Entry 2) evaluated a polymer brush (a type of polymer characterized by the presence of long polymer chains covalently attached to a surface and chains mobile) made of poly [N-(2-hydroxypropyl) methacrylamide] (poly(HPMA)) aiming to prevent mineral encrustation and the formation of *E. coli* biofilm on catheter’s surface. The surface characterization was done by Contact angle measurement, AFM, SEM and measurement of the average roughness (Ra). The efficacy of the poly(HPMA) brush as an antimicrobial coating was analyzed using synthetic urine as a growth medium in a parallel plate flow chamber (PPFC). Moreover, the authors demonstrated a reduction up to 87% and that this process makes biofilms already formed more susceptible to the action of antibiotics.

Kisuk and co-authors [40] (Table 2, Entry 3) reported the functionalization of a set of polymers such as polyvinyl chloride (PVC, polyurethane (PU), and polydimethylsiloxane (PDMS) (used to preparation of urinary catheter) with chitosan, hyaluronic acid, and human serum albumin (in some cases impregnated with silver nanoparticles) and tested against *E. coli* and *S. aureus*. According to the authors, these functionalized polymers were characterized by SEM, contact angle measuring, scanning probe microscope (SPM), XPS and UV-Vis. The authors also observed a reduction in bacterial adhesion/biofilm formation (up to ~100%) against *S. aureus* and *E. coli* and using chitosan embedded with AgNPs on the PU polymer. Moreover, the authors showed that the coating’s antimicrobial properties can be modulated by adding antibacterial metallic nanoparticles (e.g., Ag^+^).

Costa and co-authors (Table 2, Entry 4) developed a marine cyanobacterial polymer-based coating (CyanoCoating) for urinary catheter and evaluated against a set of microorganisms (*P. mirabilis*, *E. coli*, MRSA, *K. pneumoniae*, and *Candida albicans*) [41]. The CyanoCoating was characterized by AFM, water contact angle (captive bubble method), SEM and EDAX. According to the authors, this coating showed a decrease on microbial adhesion up to 68 ± 28% for *P. mirabilis*; 95 ± 48% for *K. pneumoniae*; 80 ± 27% for *S. aureus (MRSA)*; 69 ± 19% for *C. albicans*. A reduction on biofilm formation also was observed (up to 60% for *E. coli*, *P. mirabilis*, and *C. albicans*). Moreover, tests were performed using artificial urine, demonstrating a reduction of 65 ± 28% (*E. coli*), 98 ± 54% (*K. pneumoniae*), 95 ± 34% (*S. aureus-MRSA*) and 100% (*C. albicans*). 

In 2020, Alves and co-authors [42] (Table 2, Entry 5) developed a poly [oligo(ethylene glycol) methyl ether methacrylate] (poly(MeOEGMA)) as an antimicrobial coating against *E. coli*. The authors performed the characterization of coated-catheter by OCT, and SEM. After 24 h, the poly(MeOEGMA) brush reduced by 57% the adhesion of *E. coli* and, adding ampicillin obtained a reduction up to 88% for *E. coli*.

Brill and collaborators [43] (Table 2, Entry 6) applied a solution of 0.02% polyhexanide onto urethral catheter and evaluated its antibacterial efficiency against *E. coli*, *P. mirabilis,* and methicillin-resistant *S. aureus* (MRSA). A reduction of 1.64 log_10_ (swab extraction) and 2.56 log_10_ for membrane filtration compared to the control catheter were observed. The swab extraction and membrane filtration methods are based on use of a sterile cotton swab to collect the bacteria from the inner lumen of the catheters and the application of a solution to extract bacteria of the catheter surfaces followed by microbial count, respectively. Moreover, the authors did not show any characterization data regarding the presence of polyhexanide on the catheter surface.

Khandwekar and co-authors (Table 2, Entry 7) reported a study of the anti-fouling capacity and effectiveness of polyurethane polymer modified with a polyvinylpyrrolidone complex (PVP-I) (Tecoflex^®^) against *S. aureus* and *P. aeruginosa* [44]. The functionalized-catheter was characterized by FTIR, AFM and SEM-EDAX and results showed an adhesion reduction of 86% for *S. aureus* and 80% for *P. aeruginosa*. Furthermore, the lubricity of the polymer modified with (Tecoflex^®^) was analyzed, demonstrating a greater lubricity in comparison with the non-adapted polyurethane. 

A group of researchers [45] (Table 2, Entry 8) reported the functionalization of urinary catheter with methoxylated polyethylene glycol 3,4-dihydroxyphenylalanine (DOPA) plus AgNO_3_ or NaIO_4_ and its characterization by contact angle analysis, and testing against *E. coli*. The authors described that the best results were obtained using DOPA polymer with 0.25 mg/mL AgNO_3_, showing a reduction of 99.9% for *E. coli*. Moreover, the authors reported that the concentration used for AgNO_3_ is safe for application in humans.

In 2018, Raut and co-authors [21] (Table 2, Entry 9) reported the use of polyvinylpyrrolidone–iodine complex (PVPI) as coating for polyurethane (PU). This coated-polymer was applied against *S. aureus*, *S. epidermidis* and *P. aeruginosa* and characterized by contact angle, FTIR, CSLM, SEM-EDAX, TGA, and DSC. Antimicrobial tests were done using different concentrations of PVPI from 0.5 to 1.5% p/p in the PU/PVPI blends and a progressive increase of antimicrobial activity to the increase of PVPI concentration was observed. The PVPI concentration of 1.5% w/w demonstrated the best results, reducing the bacterial adhesion up to 99.8% for *S. aureus*, 99.0% for *S. epidermidis* and 89.0% for *P. aeruginosa*.

Mahata and co-authors [46] (Table 2, Entry 10) complexed N-glycidyl histidine ether with tannic acid followed by its functionalization onto the urinary catheter surface. This new catheter was characterized by a set of techniques (described in Table 2, entry 10) and a bacterial adhesion reduction (up to 90%) was observed for clinically isolated uropathogen *E. coli*. Wang and co-authors [47] (Table 2, Entry 11) reported the functionalization of silicone urinary catheter surface pre-treated with polydopamine (PDA) with a carboxymethyl chitosan derivative (CMCS) and its application against *E. coli* and *P. mirabilis*. The authors observed 90% of reduction for *P. mirabilis* and *E. coli* biofilm adhesion.

### 2.4. Silver Salts

Silver (Ag^+^) presents a long history as an antimicrobial agent [48]. In this regard, silver (e.g., salt form, colloidal, nanoparticles, and other pharmaceutical formulations) has been used as an antiseptic for surgical procedures, wound treatment, dentistry, water purification, and in medical devices surfaces. Regarding its toxicity, there are some studies describing that Ag^+^ is toxic against mammalian cells (in vitro) but research is needed to evaluate its toxicity in a controlled and randomized clinical study [49,50]. Due to its efficiency against a vast group of pathogenic microorganisms, some authors promoted its functionalization onto medical devices such as urinary catheters. Table 3 shows a description of functionalized urinary catheters with silver (2011–2021) and their evaluation parameters used for characterization, microorganisms, and efficiency.

Kumar and co-authors [51] (Table 3, Entry 1) reported the use of Kocuran, an exopolysaccharide produced by *Kocuria rosea* strain BS-1 to synthesize silver glyconanoparticles by a green protocol. The functionalized medical device was characterized by SAED, colloidal stability at different pH, CSLM, and SPR. This functionalized catheter was evaluated against *S. aureus* and *E. coli* showing an inhibition of biofilm development up to 90%. Moreover, the authors evaluated the cytotoxicity of the functionalized catheter using human gingival fibroblasts presenting a low toxicity.

Evliyaoglu and co-authors [52] (Table 3, Entry 2) reported the synthesis of Ag-incorporated nano-HA coated urinary catheters and their evaluation against prophylaxis of biofilm formation and also bacteriuria using rabbit models (3, 5 and 7 days of the urethral catheterization time in rabbits). The authors described the evaluation of a control group (siliconized latex-based urethral catheters) for comparison with the functionalized urinary catheter. For bacterial analysis, urine and catheter surface were used for quantification of efficiency of the coated catheter. Concerning the results obtained, the authors showed that at the end of 7 days (catheterization protocol), the number of the rabbits with the functionalized catheter bacteriuria was significantly lower when compared to the control group.

Wang and co-authors [53] (Table 3, Entry 3) developed a nanocomposite of silver-polytetrafluoroethylene (Ag-PTFE) as coating and its deposition on a silicone urethral catheter surface. This coated-catheter was tested against *E. coli* and anti-encrustation performances against *P. mirabilis* and characterized by SEM, EDX and contact angle measurement. To evaluate this antimicrobial coating, two concentrations of bacterial suspension challenged each model and results were used and demonstrated an increase in the time to begin biofilm formation compared to the control group (6 to 41 days).

Wang and co-authors [22] (Table 3, Entry 4) developed a coating for urinary catheter using polydopamine (PDA) and silver nanoparticles (AgNPs) and promoted its characterization using FESEM, XPS analysis, CLSM, spread plate method and contact angle measurement. The coated-catheter was tested against *E. coli*, *P. aeruginosa* and *P. mirabilis*. Results demonstrated that the release of silver depends on the number of PDA-AgNPs bilayers on the modified catheter surface. One AgNP-PDA by layer is capable of resisting encrustation for 12 days, while two bilayers in combination with poly(SBMA-co-AAm) as a final graft layer could resist up to 45 days and reduced by 99% the adhesion of bacteria on catheter surface.

The catheters immobilized with silver salts should be functionalized with efficient protocols due to the leaching of silver from the catheters. This leaching can produce an accumulation and consequent in vivo toxicity. In vitro and in vivo experiments should be performed using analytical techniques to analyze it.

### 2.5. Antimicrobial Derivative Compounds, Bacteriophages, and Enzymes

Besides the investigation of aPDT, antibiotics, antibacterial polymers, and silver salts as antimicrobial coating for urinary catheter, there are other strategies and compounds being applied so far. Table 4 shows a description of functionalized urinary catheters with antimicrobial compounds immobilized on nanoparticles, molecules extracted from soil, use of bacteriophages, and antimicrobial enzymes (2011–2021) and their evaluation parameters used for characterization, microorganisms, and their efficiency.

Kanugala and co-authors [54] (Table 4, Entry 1) reported the synthesis of mesoporous silica nanoparticles via base-catalyzed sol-gel process followed by immobilization of phenazine-1-carboxamide onto their surface. The nanoparticles were characterized by FT-IR, UV-vis, XRD spectroscopic techniques, DLS, SEM, TGA, TEM, and BET analysis. Regarding the antimicrobial evaluation, the phenazine-1-carboxamide functionalized mesoporous silica nanoparticles as a coating layer for ureteral catheters was evaluated against *C. albicans-S. aureus* polymicrobial biofilm. The authors described that the PCN-MSNPs-immobilized urethral catheters at a concentration of 2.23 mM showed no formation of *C. albicans-S. aureus* biofilms. However, the author did not present any evaluation concerning its toxicity.

Janek and co-authors [55] (Table 4, Entry 2) used a biosurfactant produced by *Rhodococcus fascians* BD8, isolated from Arctic soil, characterized by HPLC, and surface tension reduction and reported its antiadhesive and antimicrobial properties against several pathogenic microorganisms. Results showed a reduction of 95% of *C. albicans* and 70% for *E. coli* biofilm adhesion to a silicone and polystyrene surface. This biosurfactant presents potential to be applied as an antimicrobial coating for urinary catheter.

Lehman and co-authors [56] (Table 4, Entry 3) reported a pre-treated silicone urethral catheter with a hydrogel containing a blend of *P. aeruginosa* and *P. mirabilis* bacteriophages and tested against multiresistant bacteria (clinical isolates) in a continuous-flow in vitro model using artificial urine. The authors evaluated biofilm growth at 72–96 h and observed a reduction up to 4 log_10_ CFU/cm^2^ and >2 log_10_ CFU/cm^2^ for *P. aeruginosa* and *P. mirabilis*, respectively.

Cadavid and co-authors [57] (Table 4, Entry 4) conducted a study to demonstrate the formation of *K. pneumoniae* biofilm under the effect of several natural substances. To define which compounds would follow in the study, different concentrations of the compounds were tested for the viability of the bacteria. The compounds that reached 85% reduction in viability went on to analyze the ability to inhibit biofilm formation. The best results were obtained of 20-hydroxycinnamic acid and 3-methyl-2(5H)-furanone, which inhibited the formation of biofilm by 65.06% and 67.38%, respectively. Moreover, the authors observed alterations in the adherence and biofilm formation on the PVC-based urethral catheter by SEM.

Ivanova and co-authors [58] (Table 4, Entry 5) promoted the functionalization of a silicone urinary catheter with acylase enzyme via layer-by-layer technique (negatively charged enzyme and positively charged polyethyleneimine) to inhibit the growth of *P. aeruginosa* biofilm. The surface characterization of the acylase coated catheter was carried out by FTIR, AFM, water contact angle measurements, and fluorescence microscopy. The acylase-immobilized urinary catheter was able to reduce the *P. aeruginosa* biofilm (up to ~50%) and did not affect the viability of human fibroblasts (over 7 days).

Colleta and co-authors [59] (Table 4, Entry 6) carried out a functionalization process of silicone Foley catheter with *S*-Nitroso-N-acetylpenicillamine (SNAP) (a release nitric oxide (NO) polymer) and its application on reduction of microbial biofilm formation (*S. epidermidis* and *P. mirabilis*). The NO is widely described as a potent antimicrobial agent and vasodilator. The authors characterized the coated-catheter with UV-Vis, Sievers chemiluminescence and fluorescence microscopy and a generation of NO surface-fluxes of 0.7 × 10^−10^ mol min^−1^ cm^−2^. Concerning the antimicrobial evaluation, the best result of *S. epidermidis* was after 14 days, with a biofilm formation reduction of 3.7 log. To *P. mirabilis* the best result was also after 14 days, with a reduction of 6 log.

## 3. Conclusions and Future Perspectives

The main challenges in treating infections caused by microorganisms associated with biofilms are their challenging diagnosis and in the clinic, biofilms can also be difficult to eradicate due to high antibiotic tolerance [24]. In this context, the control of microbial biofilm-associated infections in medical devices requires that new and effective approaches be developed [17]. Strategies for the control of biofilms can be divided between the prevention of biofilm formation by inhibiting or blocking the adherence of microorganisms and the eradication of pre-formed biofilms through the use of strategies such as aPDT, nanotechnology, direct combat, electric current, and the development of antimicrobial-coated medical devices [60].

In order to address infections in the urinary tract, over the last years (2010–2021) a set of antimicrobial coatings have been prepared and applied onto the urinary catheter surface. These coatings are based on use of covalent and electrostatic strategies using photosensitizing entities for application in aPDT protocol (production of ROS), commercial antibiotics, synthetic and natural polymers, the traditional silver salt, and other antimicrobial entities such as enzymes, and bacteriophages. Regarding the antimicrobial evaluation and efficiency, the authors have evaluated these coated-urinary catheters against planktonic and biofilm (different species and composition) aiming to demonstrate the inhibition of growth and elimination (% or log unit) and satisfactory results have been described in the literature. However, different protocols for biofilm formation have been applied and a standardization on experimental protocol should be done to facilitate the comparison of the results. Concerning the safety tests for the new coated-urinary catheter, some studies have presented in vitro cytotoxicity tests using fibroblast cells showing low toxicity but more tests (e.g., animal models) should be made to prove the real safety of the new coated-urinary catheters. According to the literature and the results presented in this review paper, we propose the following perspectives for the next studies for the urinary catheters: (i) to explore the use of photodynamic action as described for others medical devices (such as endotracheal tube) [61]; (ii) combination of commercial antibiotics and antimicrobial coatings; (iii) to present a systematic safety study of the coated-urinary catheter; (iv) to promote the standardization of the biofilm formation protocols; (v) to evaluate the mechanical properties (i.e., tensile property) of the functionalized-urinary catheters; (vi) to transpose the coated-urinary catheter described in literature for in vivo evaluation (e.g., animal models); (vii) to evaluate the cost and viability of the proposal antimicrobial coatings.

In sum, those antimicrobial coatings for urinary catheters can be useful for decrease of number and severity of UTIs but the health professionals should apply the clinical guidelines for catheter management in order to prevent the catheter-associated urinary tract infections [12,62].

## Figures and Tables

**Table 1:** Antibiotics- and antimicrobial agent-functionalized urinary catheters applied against bacteria.

Entry	Antibiotics (Amount of Drug Loaded into Catheter)	Characterization Techniques	Bacteria	Results (Reduction)	Entry
1	Rifampicin, triclosan, and sparfloxacin (7.74–222.42 µg/cm^3^)	AFM, X-ray photoelectron spectroscopy (XPS), static model, In vitro flow model, and high-performance liquid chromatography (HPLC)	*E. coli*, *Methicillin-resistant Staphylococcus aureus* (*MRSA*), *Klebsiella pneumoniae*, *Proteus mirabilis*, *Staphylococcus saprophyticus*, and *Enterococcus faecalis* (clinical isolate)	Up to 100% (after 24–72 h)	[36]
2	Rifampicin, triclosan, and sparfloxacin (0.080–1.084% w/w)	XPS, AFM, TEM, HPLC Static model, in vitro flow model, and SEM	*S. saprophyticus*, *P. mirabilis*, *S. aureus* and *E. coli* (clinical isolate)	Prevention of colonization of *S. aureus*, MRSE, beta-lactamase producing *E. coli*, and arbapenemase-producing *E. coli* during 12 weeks	[19]
3	Rifampicin, triclosan, and sparfloxacin	HPLC, SEM, and time of flight secondary ion mass spectrometry (ToF-SIMS)	*S. aureus* and *E. coli* (clinical isolate)	100% of attached *E. coli* (tK100)	[20]

**Table 2:** Antimicrobial polymers applied against microorganisms and the parameters used.

Entry	Polymers (Amount of Polymer onto Catheter)	Characterizations	Microorganisms	Results (Reduction) % or Log Unit	Ref
1	Colloidal polysaccharides immobilized on silicone	Fourier-transform infrared spectroscopy (FTIR), SEM, XPS, confocal laser scanning microscopy (CLSM)	*S. aureus*, *E. coli*, *MRSA*, and *C. albicans* (clinical isolate)	Inhibition of bacterial growth up to 86% (incubation for 18 h)	[38]
2	Poly[N-(2-hydroxypropyl) methacrylamide] (poly(HPMA))	Contact angle measurement, measurement of the average roughness (Ra), AFM, and SEM	*E. coli* (CECT 434 and clinical isolate)	Reduction up to 87% (biofilm formation for 24 h)	[39]
3	Catechol-based hydrogel film	SEM, XPS, contact angle measuring, and ultraviolet–visible spectroscopy (UV-Vis)	*E. coli* (ATTC 29906) and *S. aureus* (ATTC 6341)	Reduction on bacterial adhesion/ biofilm formation (8 h) (up to 100%)	[40]
4	Cyanobacterial polymer-based coating	AFM, SEM, and water contact angle	*E. coli* (ATCC 25922), *MRSA* (ATCC 33591)*, K. pneumoniae* (clinical isolate)*,* and *C. albicans* (DSM 1386)	Reduction of adhesion up to 100% for *C. albicans* and biofilm formation (12 h) (*E. coli*) up to 60%	[41]
5	Poly[oligo(ethylene glycol) methyl ether methacrylate], poly(MeOEGMA)	SEM, and Optical Coherence Tomography (OCT)	*E. coli* JM109(DE3)	Reduced the adhesion of *E. coli* (up to 57%); ampicillin reduced up to 88% of *E. coli* (up to 24 h)	[42]
6	0.02% polyhexanide solution	Characterization was not performed	*S. aureus* (*ATCC 6538*), *Enterococcus hirae* (*ATCC 10541*), *E. coli K12* (*ATCC 11229*), *P. mirabilis* (*ATCC 14153*), *P. aeruginosa* (*ATCC 15442*), and *K. pneumoniae* (*ATCC 16609*)	Reduction of 1.64 log_10_ (incubated for 72 h)	[43]
7	Polyvinylpyrrolidone-iodine engineered polyurethane (Tecoflex^®^) (90 ± 4 µg/cm^2^)	FTIR, AFM, SEM- energy dispersive analysis of X-rays (EDAX), and water contact angle measurements	*S. aureus* (NCIM 5021) and *P. aeruginosa* (NCIM 5029)	Adhesion reduction: *S. aureus* (by 86%;) and *P. aeruginosa* (80%) in 4 h	[44]
8	Methoxylated polyethylene glycol 3,4-dihydroxyphenylalanine (DOPA) copolymer with AgNO_3_ or NaIO_4_	Contact angle analysis	Uropathogenic clinical isolates, and *E. coli* (CFT073)	Showed a 99.9% bacterial killing	[45]
9	Polyurethane (PU) blends with polyvinylpyrrolidone iodine (PVPI) (0.5–1.5% w/w)	CLSM, contact angle, FTIR, SEM-EDAX, thermogravimetric analysis (TGA), and differential scanning calorimetry (DSC)	*P. aeruginosa* (NCIM 5029), *S. aureus* (MCC 2408), and *S. epidermidis* (NCIM 2493)	Reduction of the bacterial adhesion up to 99.85% for *S. aureus* (4–48 h)	[21]
10	N-glycidyl histidine ether with tannic acid (solution applied of 1 mg/mL)	UV-Vis, FTIR, nuclear magnetic resonance spectroscopy (NMR), TGA, SEM, TEM, AFM, and dynamic light scattering (DLS)	Clinically isolated uropathogen, *E. coli*	Reduction of the bacterial adhesion up to 90% (biofilm growth for 7 days)	[46]
11	Carboxymethyl chitosan on medical grade silicone surface pre-treated with polydopamine (PDA)	XPS, and contact angle measurements	*E. coli* (ATCC DH5a) and *P. mirabilis* (ATCC 51286)	Reduced the adhesion of *E. coli* and *P. mirabilis* by 90%	[47]

**Table 3:** Silver-functionalized urinary catheters applied against microorganisms.

Entry	Coating	Characterization Techniques	Microorganisms	Results (Reduction) % or Log Unit	Ref
1	Silver glyconanoparticles (AgNPs) using Kocuran, an exopolysaccharide produced by *Kocuria rosea* strain BS-1	Surface plasmon resonance (SPR), X-ray diffraction analysis (XRD), selected area (electron) diffraction (SAED), and colloidal stability at different pH	*S. aureus* (ATCC 29213) and *E. coli* (ATCC 35218)	Inhibition of biofilm formation (up to 90%)	[51]
2	Ag^+^-incorporated nano- hydroxyapatite	SEM, energy dispersive X-ray analysis (EDX)	*E. coli*, *Staphylococcus* species, *P. mirabilis*, *Enterobacter cloacae*, *P. aeruginosa*, *Pseudomonas alcaligenes*, and *A. haemolyticus* (clinical samples presented during the catheterization)	The number of the rabbits with bacteriuria was lower for 7 days of catheterization protocol	[52]
3	Ag^+^-polytetrafluoroethylene nanocomposite deposited on silicone catheters (up to 145 mm^2^/mL of ratio of catheter surface area/volume of coating solution)	EDX, contact angle values and surface energies	*E. coli* (F1693) and *P. mirabilis* (ATCC 51286)	Development of bacteriuria (10^2^ cells/mL) was an average 6 days vs. 41 days for uncoated and coated catheter, respectivelly (biofilm formation for 48 h)	[53]
4	Silver nanoparticles immobilized on polydopamine (PDA) (concentration of silver on surface: 10.2–13.2 ± 0.5–0.8 µg/cm^2^)	Field emission scanning electron microscopy (FESEM), XPS, and contact angle	*E. coli*, *P. mirabilis*, and *P. aeruginosa*	Encrustation up to 45 days	[22]

**Table 4:** Functionalization of urinary catheters applied against microorganisms.

Entry	Coating	Characterization Techniques	Microorganisms	Results (Reduction) % or Log Unit	Ref
1	Mesoporous silica nanoparticles functionalized with phenazine-1-carboxamide (coated with 500 µg mL^−1^ of nanoparticles)	UV-vis, FTIR, DLS, XRD, SEM, TEM, TGA, and Brunauer-Emmett-Teller (BET) surface area analysis (BET)	Polymicrobial biofim (*C. albicans* (MTCC 227)*-S. aureus* (MTCC 96))	No formation of polymicrobial biofilm (incubation times of 24, 48, and 72 h)	[54]
2	Trehalose lipid biosurfactnt from *Rhodococcus fascians* BD8 (Arctic soil) (0.035–0.5 mg mL^−1^)	HPLC, and surface tension reduction	*C. albicans* (ATCC 10231, and SC5314) and *E. coli* (clinical isolate, ATCC 10536, and ATCC 25922)	95 and 70% antiadhesive activity against *C. albicans* and *E. coli*, respectively (incubated for 2 h on polymer surface)	[55]
3	Hydrogel with a mixtures of *P. aeruginosa* and *P. mirabilis* bacteriophages	-	*P. aeruginosa* and *P. mirabilis* (clinical isolates)	Reduction up to 4 log_10_ and >2 log_10_ for *P. aeruginosa* and *P. mirabilis*, respectively (biofilm growth for 72 to 96 h)	[56]
4	Natural derivative substances (phenyl-acyl derivatives, pyridines, pyrroles, pyrazines, and pyrans) (Concentration at 15 µg/mL)	SEM	*K. pneumoniae* (ATCC 13884)	Hydroxycinnamic acid derivative inhibited the formation of biofilm up to 67.38% (incubated times of 6 and 24 h)	[57]

## Data Availability

Not applicable.
